# Associations of Dietary Patterns and Vitamin D Levels with Iron Status in Pregnant Women: A Cross-Sectional Study in Taiwan

**DOI:** 10.3390/nu15081805

**Published:** 2023-04-07

**Authors:** Arpita Das, Chyi-Huey Bai, Jung-Su Chang, Ya-Li Huang, Fan-Fen Wang, Yi-Chun Chen, Jane C.-J. Chao

**Affiliations:** 1 School of Nutrition and Health Sciences, College of Nutrition, Taipei Medical University, 250 Wu-Hsing Street, Taipei 110301, Taiwan; 2 Department of Public Health, School of Medicine, College of Medicine, Taipei Medical University, 250 Wu-Hsing Street, Taipei 110301, Taiwan; 3 School of Public Health, College of Public Health, Taipei Medical University, 250 Wu-Hsing Street, Taipei 110301, Taiwan; 4 Nutrition Research Center, Taipei Medical University Hospital, 252 Wu-Hsing Street, Taipei 110301, Taiwan; 5 Graduate Institute of Metabolism and Obesity Sciences, Taipei Medical University, 250 Wu-Hsing Street, Taipei 110301, Taiwan; 6 Department of Metabolism, Yangming Branch, Taipei City Hospital, 105 Yusheng Street, Taipei 111024, Taiwan; 7 Master Program in Global Health and Health Security, Taipei Medical University, 250 Wu-Hsing Street, Taipei 110301, Taiwan; 8 TMU Research Center for Digestive Medicine, Taipei Medical University, 250 Wu-Hsing Street, Taipei 110301, Taiwan

**Keywords:** vitamin D, iron, dietary pattern, principal component analysis, gestational anemia

## Abstract

Vitamin D is involved in the pathophysiology of anemia. This cross-sectional study was conducted using the Nationwide Nutrition and Health Survey in Pregnant Women in Taiwan database. We investigated associations among dietary patterns (DPs), vitamin D, and iron-related biomarkers in pregnant women. The principal component analysis revealed four DPs. Linear and logistic regression analyses were performed to investigate the association of DPs with anemia-related biomarkers. Plant-based, carnivore, and dairy and nondairy alternatives DPs were positively associated with serum vitamin D levels. After adjusting covariates, the pregnant women consuming plant-based DPs at the mid-tertile (T2) were associated with reduced risks of low serum folate and vitamin D levels, and those consuming carnivore DPs at higher tertiles (T2 and/or T3) were correlated with an increased risk of low serum iron levels but decreased risks of low serum transferrin saturation, vitamin B_12_, and vitamin D levels. The pregnant women consuming dairy and nondairy alternatives DPs at the highest tertile (T3) were associated with reduced risks of low serum folate and vitamin B_12_ levels. However, the processed food DP was not correlated with anemia-related biomarkers. Thus, plant-based, carnivore, and dairy and nondairy alternatives DPs were associated with the risk of low-serum-anemia-related variables.

## 1. Introduction

Anemia during pregnancy or gestational anemia is a major health concern affecting approximately 38% of the global population (approximately 32 million individuals); this proportion ranges from 24% in the Western Pacific Region to 49% in Southeast Asia [1,2]. The World Health Organization (WHO) has defined anemia as a hemoglobin (Hb) level of <6.83 mmol/L (<11 g/dL) and severe anemia as an Hb level of <4.34 mmol/L (<7 g/dL) [3]. For pregnant women, the trimester-wise classification proposed by the Center for Disease Control and Prevention (CDC) suggests that gestational anemia can be indicated by an Hb level of <6.83 mmol/L (<11 g/dL) in the first and third trimesters and that of <6.52 mmol/L (<10.5 g/dL) in the second trimester [4].

Gestational anemia increases the incidence rates of perinatal mortality, stillbirth, abnormal or retarded brain growth, and fetal morbidity [5,6]. Iron deficiency anemia is the most common type of gestational anemia and indicated by a serum ferritin level of <0.034 nmol/L (<15 µg/L) [7]. Other common causes of gestational anemia include folate (megaloblastic anemia) or vitamin B_12_ (pernicious anemia) deficiency, which contributes to maternal morbidities [8,9]. Fetal nutrient deficiencies may result from congenital abnormality, low birth weight, and preterm delivery [10,11]. Iron is a key micronutrient essential for tissue oxygenation and erythropoiesis. Blood loss, decreased iron intake, and impaired iron absorption could contribute to iron deficiency [12]. Gestational iron storage and the absorption of dietary iron are important for the maintenance of iron homeostasis. Ferritin is a protein form which stores iron and serves as a preliminary predictor of lower hemoglobin and anemia [13,14]. Hence, in the present study, the major variables related to anemia were ferritin followed by hemoglobin and serum iron levels. In a study that took place in the UK and Australia, a serum ferritin test in the first trimester was suggested to verify whether pregnant women needed to be referred for iron therapy, and serum ferritin levels were considered to be assessed in the first antenatal visit for women from areas with a high prevalence of iron-deficiency anemia, along with a full blood count test in early pregnancy for women at high risk of iron-deficiency anemia [15]. Additionally, a prospective cohort study of maternal and infant health and nutrition surveillance in Bangladesh determined maternal plasma ferritin levels at gestational weeks 14 and 30 and found that plasma ferritin levels in the late gestation of pregnancy were negatively correlated with infant birth weight [16], indicating the crucial role of ferritin as a form of iron storage in fetal growth outcome.

Several dietary nutrients affect iron balance, and the antioxidant vitamin C, as an acidic substance, promotes iron absorption [17]. Most earlier studies have focused on the role of vitamin C in dietary iron absorption [18,19]. However, few studies have explored the association between dietary patterns (DPs) and vitamin D levels in women with gestational anemia. Iron absorption was reportedly enhanced by vitamin D through reducing the levels of hepcidin and proinflammatory cytokines [20,21]. However, the role of vitamin D in anemia prevention and iron absorption remains controversial [22]. In animal- and population-based pregnancy studies, Qiu et al. [23] and Si et al. [24] both reported a positive association between blood vitamin D levels and iron status. A cross-sectional study conducted by Mayasari et al. revealed an association between dietary intake and serum hepcidin levels during pregnancy [25]. Furthermore, an evidence-based study conducted by Michalski et al. among Vietnamese women of reproductive age reported a positive association between serum, instead of dietary, vitamin D and Hb levels [26]. Additionally, Wong et al. found that serum vitamin D levels were positively associated with serum ferritin levels in Chinese pregnant women [27]. However, the aforementioned studies did not explore any other iron-related biomarkers. Our knowledge regarding DPs, vitamin D levels, and iron status remains limited. In the present study, DP was used as a supportive approach to investigate the association between overall dietary factors and disease outcomes [28]. Thus, we investigated the association of DPs with vitamin D levels and other iron-related biomarkers in pregnant women.

## 2. Materials and Methods

### 2.1. Study Design and Population Demographics

This population-based cross-sectional study was conducted using a database associated with the Nationwide Nutrition and Health Survey in Pregnant Women in Taiwan (2017–2019; Pregnant NAHSIT 2017–2019). Relevant data were collected from a total of 11 recognized hospitals in Taiwan. The inclusion criteria were as follows: being aged >15 years; receiving a maternal handbook; using an obstetric inspection service more than once; being able to communicate in Mandarin, Taiwanese, and other languages; and being willing to participate in our study and provide consent. The exclusion criteria included having multiparity (>3) and being nonresponsive.

The data of 1502 pregnant women were used in the present study. After the participants signed the consent form, the researchers assigned the date for collecting data during their prenatal visits. The collected data were classified into the following four categories: sociodemographic, anthropometric, biochemical, and dietary (including supplements, such as milk powder, multivitamin/multimineral, iron, vitamin B complex, folate, vitamin D, and calcium, and dietary assessment) data. Sociodemographic and anthropometric data were obtained using a self-reported questionnaire, whereas dietary data were collected by well-trained registered dieticians during face-to-face interviews with the women. The data collection from all the questionnaires took 60–90 min. Biochemical analyses were performed using blood samples collected during prenatal visits. This study was approved by the Joint Institutional Review Board of Taipei Medical University, Taiwan (approval number: TMU-JIRB N201707039) and conducted in accordance with the ethical principles of the Declaration of Helsinki.

### 2.2. Dietary Assessment

Dietary assessment was performed using a 24 h dietary recall method and a validated semiquantitative food frequency questionnaire (FFQ) modified from the NAHSIT FFQ [25]. Food pictures and measurement cups or spoons were used when 24 h dietary recall was conducted by registered dieticians. The FFQ is the most commonly used, reliable, and cost-effective tool for nutrition surveys and has high reproducibility [29]. A total of 59 food items were identified using the FFQ. For the present study, a total of 32 food groups were developed based on the categories and nutrient contents of the aforementioned food items [25]. Food items having similar nutrient characteristics were categorized under the same group (Supplementary Table S1).

The daily, weekly, or monthly frequencies of food intake were recorded in the FFQ. The total monthly frequency of a particular food group was calculated. According to the 24 h dietary recall data, nutrient intake was calculated using Cofit Pro (Cofit Healthcare, Taipei, Taiwan), an online software available on the Taiwan Food Nutrient Database.

DPs can be determined using two approaches: a priori (a hypothesis-derived prospective study) and a posteriori (a data-driven, frequency-based retrospective study) methods [30]. Principal component analysis (PCA) was performed in the present study to determine the DPs of the women, because PCA (an a posteriori method) offered the highest interpretability with minimal information loss and reduced dataset dimensionality [31].

### 2.3. Anthropometric and Biochemical Data Collection

Pre-pregnancy body mass index (BMI) was calculated using body weight (kg) divided by height (m^2^). Both body weight and height before pregnancy were self-reported and collected in the questionnaire. Blood was drawn from the median cubital and cephalic veins. Serum hemoglobin (Hb) levels were measured using a hematology analyzer (Sysmex Corp., Kobe, Japan). Serum iron levels (µmol/L) were determined spectrophotometrically using a Beckman Coulter Unicel DxC 800 (Beckman Coulter, Brea, CA, USA) after iron was released by acetic acid from transferrin and reduced to ferrous iron by hydroxylamine and thioglycolate [25]. Serum ferritin levels were assessed by an enzyme-linked immunosorbent assay using the Beckman Coulter Unicel DxC 800 (Beckman Coulter, Brea, CA, USA) [25]. The total iron-binding capacity (TIBC, μmol/L) was evaluated by the colorimetric immunoassay method using the Beckman Coulter Unicel DxC 800 (Beckman Coulter, Brea, CA, USA) [32]. Transferrin saturation (%) was calculated by the percentage of serum iron levels divided by the TIBC value [33]. The serum levels of folate [34] and vitamin B_12_ [35] were measured using SimulTRAC-SNB radioimmunoassay kits (MP Biomedicals, Santa Ana, CA, USA) with ^125^I or ^57^Co as the tracer, respectively. Serum 25(OH) vitamin D levels were determined by an electrochemiluminescence immunoassay using an Elecsys vitamin D total reagent kit with ruthenium-labeled vitamin-D-binding protein (Roche Diagnostics Ltd., Taipei, Taiwan) [36].

### 2.4. Anthropometric and Biochemical Parameters in Gestational Anemia

The Ministry of Health and Welfare, Taiwan, has recommended the following BMI-based classification of adults: underweight (<18.5 kg/m^2^), normal weight (18.5 to <24 kg/m^2^), overweight (24 to <27 kg/m^2^), and obesity (>27 kg/m^2^) [37]. Gestational anemia was defined according to the criteria outlined by the WHO and CDC. The normal cutoff values of serum iron and TIBC in women without anemia are 10.7 µmol/L (60 µg/dL) [38] and 42.96–80.55 µmol/L (240–450 µg/dL) [39]. The WHO recommends the following cutoff values for gestational anemia: serum ferritin level <0.034 nmol/L (<15 µg/L) [40] and transferrin saturation <16% [41]. The reference levels of serum folate for all age populations are 13.6–45.3 nmol/L (6–20 ng/mL) [42]. The Endocrine Society has defined vitamin D insufficiency as a vitamin D level of <75 nmol/L (<30 ng/mL) [43].

### 2.5. Statistical Analysis

Statistical analysis was performed using SPSS (version 22.0, IBM Corp., Armonk, NY, USA) and SAS (version 9.4, SAS Institute Inc., Chicago, IL, USA). A one-way analysis of variance was used for continuous variables expressed as mean ± standard deviation, whereas the chi-square test was used for categorical variables expressed as number and percentage. Tukey’s post hoc multiple comparisons were performed to determine significant within-group differences among continuous variables. We identified DPs by PCA using SAS. A total of four DPs were identified through orthogonal varimax rotation with a mean eigenvalue of 1.0 and a factor loading of >0.30 [44]. Factor loadings of <0.30 were omitted for simplification. A high factor loading indicates a strong association between food groups and disease. For each DP, DP scores were calculated by total food intake (frequency/month) times factor loading. We used the following three models to verify the association between DPs and blood biomarker levels: model 1 (crude model), model 2 (adjusted for age, region of residence, parity, and trimester), and model 3 (adjusting factors in model 2 plus daily dietary intake). A simple linear regression analysis was conducted using the independent (DP) and dependent (biochemical biomarkers) variables to identify the trend (positive or negative) of association. Data are presented in terms of the regression of coefficient (β) and 95% confidence intervals (CIs). For further confirmation, each DP was categorized into tertiles. Tertiles 1 (T1), 2 (T2), and 3 (T3) represent the lowest, mid, and highest DP scores, respectively. Furthermore, a binomial logistic regression analysis was performed to identify the disease trend across the tertiles of each DP and biochemical biomarkers, and the odds ratios (ORs) of T2 and T3 were compared with the reference group (T1). Data are presented in terms of odds ratios and 95% CIs. The OR value of >1 or <1 with statistical significance indicates increased or decreased disease risk, whereas OR = 1 represents nonsignificant effects [45]. Statistical significance was set at *p* ≤ 0.05.

## 3. Results

### 3.1. Sociodemographic and Anthropometric Characteristics of the Women

Pregnant women in T3 of serum vitamin D were older (32.9 ± 4.9 vs. 32.0 ± 4.7 years) than those in T1 of serum vitamin D (Table 1). Most pregnant women in T3 of serum vitamin D lived in the southern part of Taiwan (32%), were primiparous (49.3%), and were in the third trimester of pregnancy (53%). The women across the vitamin D tertiles did not differ significantly in terms of education level, family monthly income, duration of sun exposure, or BMI.

### 3.2. Biochemical Characteristics of the Women

Pregnant women in T3 of serum vitamin D had higher levels of serum Hb (7.4 ± 1.3 mmol/L), iron (13.9 ± 7.8 µmol/L), TIBC (85.6 ± 17.1 µmol/L), folate (32.3 ± 17.0 nmol/L), and vitamin B_12_ (249.0 ± 169.8 pmol/L), but lower serum ferritin levels (0.05 ± 0.05 nmol/L) than those in T1 of serum vitamin D did (Table 2). Categorical classification revealed that the levels of serum Hb and folate were >6.76 mmol/L and ≥13.5 nmol/L, respectively, in most women in T3 of serum vitamin D. The number of individuals with anemia defined as Hb <6.83 mmol/L (<11 g/dL) in trimesters 1 and 3 or Hb <6.52 mmol/L (10.5 g/dL) in trimester 2 was 322 (21.4%), and we did not further analyze the data based on the pregnant women with or without anemia due to there being much fewer women with anemia compared with those without anemia.

### 3.3. Dietary Intake of the Women

Daily dietary intakes of energy, macronutrients, iron, folate, vitamin B_12_, and vitamin D were determined using 24 h dietary recall data. Pregnant women in T3 of serum vitamin D had higher intakes of protein (g), fat (g and % of energy), iron, folate, and vitamin D, but lower intakes of carbohydrates (% of energy) than those in T1 of serum vitamin D did (Supplementary Table S2). No significant differences were found in pregnant women across the tertiles of serum vitamin D in terms of energy or vitamin B_12_ intake.

Pregnant women in T3 of serum vitamin D had higher monthly intake frequencies for supplements of multivitamin/multimineral, folate, and calcium than those in T1 of serum vitamin D did (Supplementary Table S2). Other dietary supplements such as milk powder (17.6%), iron (11.2%), vitamin B complex (18.0%), and vitamin D (11.1%) were not assessed for the monthly intake frequency because a lower proportion (<20%) of the women took these supplements.

### 3.4. Dietary Patterns

**Figure 1:**
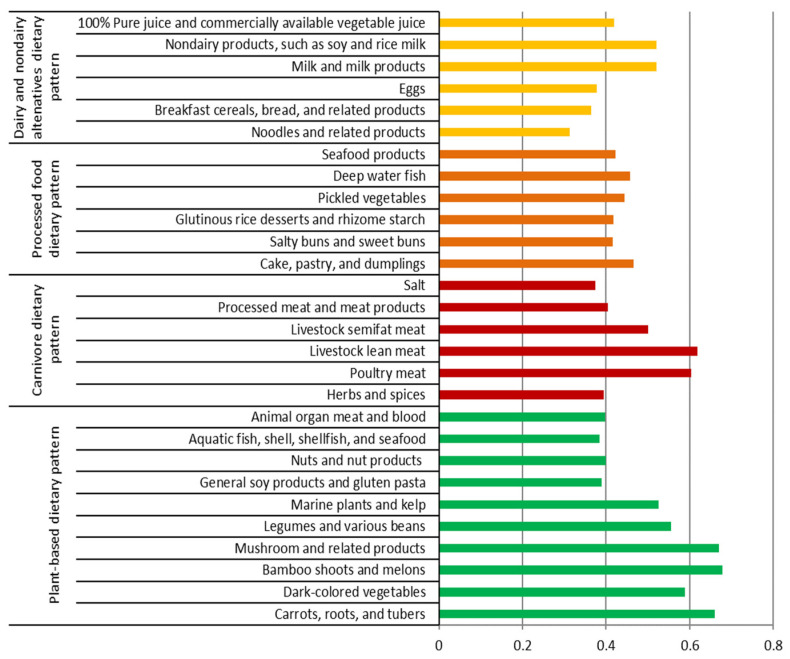
Factor loading of four dietary patterns identified by principal component analysis. The factor loadings of <0.30 were eliminated for simplification.

The PCA revealed a total of four DPs (Figure 1). All four DPs explained total variance of 9.35% (4.37%, 1.93%, 1.61%, and 1.44%). DPs were categorized and ranked on the basis of a threshold factor loading value (>0.30). Each DP was named according to their corresponding factor loading values and dietary component structures. The first pattern comprising a total of ten food groups was named the plant-based DP (DP-1) because the highest factor loadings were exhibited by bamboo shoots and melons; mushroom and related products; carrots, roots, and tubers; dark-colored vegetables; and legumes and various beans. Other food groups in DP-1 included marine plants and kelp; nuts and nut products; animal organ meat and blood; general soy products and gluten pasta; and aquatic fish, shell, shellfish, and seafood. The second pattern was named the carnivore DP (DP-2), which comprised the following six food groups from the highest to the lowest factor loadings: livestock lean meat; poultry meat; livestock lean meat; processed meat and meat products; herbs and spices; and salt. The third pattern was named the processed food DP (DP-3), which comprised the following six food groups: cake, pastry, and dumplings; salty buns and sweet buns; glutinous rice desserts and rhizome starch; pickled vegetables; deep water fish; and seafood products. Finally, the fourth pattern was named the dairy and non-dairy alternatives DP (DP-4), which comprised the following six food groups: milk and milk products; nondairy products, such as soy and rice milk; eggs; breakfast cereals and bread and related products; noodles and related products; and 100% pure juice and commercially available vegetable juice.

### 3.5. Association of DPs with Serum-Anemia-Related Biochemical Variables

Table 3 presents the association between plant-based DP (DP-1) and anemia-related biochemical variables. Serum ferritin levels in the crude model (model 1) were negatively (β: −0.06, 95% CI: −0.29, −0.01, *p* ≤ 0.05) associated with DP-1, but after covariate adjustment, there was no significant association between serum ferritin levels and DP-1. In contrast, serum TIBC in model 1 (β: 0.09, 95% CI: 0.02, 0.10, *p* ≤ 0.001) and serum vitamin D levels in all three models (model 1: β: 0.08, 95% CI: 0.02, 0.08, *p* ≤ 0.01; model 2: β: 0.06, 95% CI: 0.00, 0.06, *p* ≤ 0.05; model 3: β: 0.04, 95% CI: −0.00, 0.05, *p* ≤ 0.05) were positively associated with DP-1.

As shown in Table 4, in all the three models, carnivore DP (DP-2) was correlated with the reduction in serum iron levels by 0.07–0.08 µmol/L (model 1: β: −0.08, 95% CI: −0.49, −0.10, *p* ≤ 0.01; model 2: β: −0.07, 95% CI: −0.47, −0.07, *p* ≤ 0.01; model 3: β: −0.08, 95% CI: −0.50, −0.11, *p* ≤ 0.01). In addition, DP-2 was associated with the decrease in serum ferritin levels by 0.06 nmol/L (95% CI: −0.46, −0.04, *p* ≤ 0.05) but the increase in serum TIBC levels by 0.08 µmol/L (95% CI: 0.02, 0.10, *p* ≤ 0.01) in model 1. Changes in serum ferritin and TIBC levels were not significant after covariate adjustment. In all three models, serum vitamin D levels were positively associated with DP-2, and the increase in serum vitamin D ranged from 0.04 to 0.08 nmol/L (model 1: β: 0.08, 95% CI: 0.02, 0.10, *p* ≤ 0.01; model 2: β: 0.06, 95% CI: 0.00, 0.08, *p* ≤ 0.05; model 3: β: 0.04, 95% CI: −0.00, 0.07, *p* ≤ 0.05).

The processed food DP (DP-3) did not exhibit any strong association with anemia-related biochemical biomarkers except vitamin B_12_ (Supplementary Table S3). Serum vitamin B_12_ levels were negatively associated with DP-3 in models 1 and 2 (model 1: β: −0.04, 95% CI: −1.44, 0.09, *p* ≤ 0.05; model 2: β: −0.05, 95% CI: −1.48, 0.02, *p* ≤ 0.05).

Table 5 presents the association between the dairy and nondairy alternatives DP (DP-4) and anemia-related biochemical variables. DP-4 was positively associated with serum TIBC in model 1 (β: 0.08, 95% CI: 0.02, 0.10, *p* ≤ 0.01). Furthermore, the serum vitamin D level was only positively associated with DP-4 in models 1 and 2 (model 1: β: 0.05, 95% CI: 0.02, 0.09, *p* ≤ 0.05; model 2: β: 0.04, 95% CI: −0.00, 0.08, *p* ≤ 0.05).

### 3.6. Association of DPs with the Risk of Low-Anemia-Related Biomarkers

As shown in Table 6, the binomial logistic regression analysis revealed that the pregnant women with the highest consumption levels (T3) of plant-based DPs (DP-1) were associated with a reduced risk of low ferritin levels (OR: 0.73, 95% CI: 0.57, 0.94, *p* ≤ 0.05) in model 1 compared with those with lower consumption levels (T1) of DP-1. However, there were no significant correlations between DP-1 and the risk of low serum ferritin levels after covariate adjustment. Additionally, the pregnant women with higher consumption levels (T2 and/or T3) of DP-1 were likely to have reduced risks of low folate and vitamin D levels compared with those with lower consumption levels (T1) of DP-1 in all the models.

As found in Table 7, the pregnant women with higher consumption levels (T3 and/or T2) of the carnivore DP (DP-2) were likely to have an increased risk of low iron levels in all the models. The pregnant women with higher consumption levels (T2) of DP-2 were associated with a decreased risk of low transferrin saturation (OR: 0.70, 95% CI: 0.54, 0.91, *p* ≤ 0.01) in model 2. T2 and T3 of DP-2 were correlated with reduced risks of low serum vitamin B_12_ and vitamin D levels in the adjusted models.

The processed food DP (DP-3) did not exhibit any prominent associations with anemia-related biochemical biomarkers except serum vitamin D levels (Supplementary Table S4). The pregnant women with higher consumption levels (T2) of DP-3 were likely to have a reduced risk of low vitamin D levels in model 1 (OR: 0.71, 95% CI: 0.53, 0.95, *p* ≤ 0.05) and model 2 (OR: 0.68, 95% CI: 0.51, 0.92, *p* ≤ 0.05).

Table 8 demonstrates the associations between the dairy and nondairy alternatives DP (DP-4) and anemia-related biochemical variables. In Model 1, the pregnant women with the highest consumption levels (T3) of DP-4 were correlated with reduced risks of low serum TIBC (OR: 0.71, 95% CI: 0.54, 0.93, *p* ≤ 0.05), low vitamin B_12_ (OR: 0.73, 95% CI: 0.54, 0.97, *p* ≤ 0.05), and low vitamin D levels (OR: 0.72, 95% CI: 0.54, 0.96, *p* ≤ 0.05). After covariate adjustment, the pregnant women with the highest consumption levels (T3) of DP-4 were associated with decreased risks of low serum folate (models 2 and 3), low vitamin B_12_ (models 2 and 3), and low vitamin D (model 2).

## 4. Discussion

### 4.1. Association of Serum Vitamin D with Other Serum-Anemia-Related Biomarkers

We showed that all anemia-related biochemical variables were significantly different across the tertiles of serum vitamin D levels in the pregnant women, except for transferrin saturation. Hence, pregnant women with higher serum vitamin D levels had higher serum Hb, iron, TIBC, folate, and vitamin B_12_ levels, which indicates better iron status. Similarly, Si et al. [24] found that plasma 25(OH) vitamin D levels were positively correlated with plasma Hb levels in each trimester of Chinese pregnant women. Additionally, Chinese pregnant women with vitamin D deficiencies (<50 nmol/L) in trimesters 1 and 2 were associated with an elevated risk of anemia compared with those without vitamin D deficiencies [24]. A cross-sectional study conducted in Vietnamese non-pregnant young women revealed that serum vitamin D levels, not dietary vitamin D intake, were positively associated with Hb levels, but not significantly correlated with anemia [26]. We also found that the pregnant women with higher serum vitamin D levels had lower serum ferritin levels, but the average ferritin levels were still within the normal range. A previous study demonstrated that serum 25(OH) vitamin D levels were not correlated with serum ferritin levels in Indonesian pregnant women in the first trimester; however, the pregnant women with insufficient (<75 nmol/L) or deficient (<50 nmol/L) 25(OH) vitamin D levels in the first trimester had a higher risk of developing anemia in the third trimester [46].

### 4.2. Association of DPs with Serum-Anemia-Related Biomarkers

Our findings from the linear regression analysis revealed that both the plant-based (DP-1) and carnivore (DP-2) DPs were negatively associated with serum ferritin levels in the crude mode, but positively correlated with serum vitamin D levels in all the models. In contrast, the processed food DP (DP-3) was negatively associated with serum vitamin B_12_ levels. The dairy and nondairy alternatives DP (DP-4) was positively correlated with serum TIBC and vitamin D levels. Consistently, our findings from the binomial regression analysis showed that both DP-1 and DP-2 were associated with a reduced risk of low serum vitamin D levels. DP-4 was correlated with decreased risks of low serum TIBC, folate, vitamin B_12_, and vitamin D levels.

Plant-based foods (non-heme iron source) are rich in fiber, phytate, oxalate, and/or polyphenols which could chelate with iron as an inhibitor of iron bioavailability, and they have less iron absorption compared with heme iron food sources [47,48,49]; thus, the plant-based DP (DP-1) could be correlated with a reduction in serum ferritin levels. Our study demonstrated that DP-1 was correlated with reduced odds of low serum folate and vitamin D levels in pregnant women. Similarly, a previous study reported that pregnant women consuming an ovo-lacto vegetarian or a low-meat diet were likely to have a lower risk of folate deficiency compared with those consuming a Western diet [50]. Additionally, pregnant women consuming a vegetarian diet had significantly higher serum 1,25-(OH)_2_ vitamin D levels compared with those consuming a nonvegetarian diet [51]. However, adults consuming a vegetarian diet or a plant-based diet were correlated with lower iron stores (lower serum ferritin levels) and a higher prevalence of anemia, probably due to the poor absorption of non-heme iron compared with those consuming a nonvegetarian diet [52,53].

Notably, the carnivore DP (DP-2) was associated with an increased risk of low serum iron levels in our study. However, a systematic review reported that the adults consuming a high intake of a carnivore/animal-based diet were positively correlated with iron status [54]. The possible reason for the association between DP-2 and low serum iron levels could be attributed to herbs and spices (such as chili paper, garlic, Thai leafy vegetables, shallot, tamarind, and turmeric) in DP-2, which are enriched in polyphenolic compounds and can hinder iron absorption by forming insoluble iron complexes [55]. We also found that DP-2 was correlated with reduced risks of low transferrin saturation, vitamin B_12_, and vitamin D levels. Norwegian women (36–39 years old) consuming a reindeer meat DP were likely to have slightly higher transferrin saturation (mean: 28%) compared with those consuming a fish (mean: 26%), average (mean: 27%), fruit/vegetables (mean: 24%), or Western/marine DP (mean: 26%) [56]. Dutch pregnant women who consumed higher vitamin B_12_ intake from animal foods such as meat, fish, or dairy food which were rich in vitamin B_12_ were correlated with higher plasma vitamin B_12_ levels [57]. A previous study showed that Caucasian pregnant women in Ireland consumed dietary vitamin D primarily from meat, eggs, and breakfast cereals [58]. Meat was the predominant food group in DP-2, and the pregnant women with higher intakes of DP-2 presumably had better serum vitamin D statuses.

The processed food DP (DP-3) was negatively associated with serum vitamin B_12_ levels. The excessive thermal treatment of foods during food processing may be attributed to reduced vitamin B_12_ content in foods [59]. Additionally, high intakes of ultra-processed foods were correlated with decreased intakes of certain vitamins such as vitamin A, B_12_, C, D, E, and niacin in adults [60].

After covariate adjustment, T3 of the dairy and nondairy alternatives DP (DP-4) was associated with reduced odds of low serum folate, vitamin B_12_, and vitamin D levels. Consistent with our findings, Cifelli et al. [61] demonstrated that dairy and individual dairy foods were correlated with increased serum folate and vitamin B_12_ levels in a US population. Dairy food also provided rich sources of vitamins B_12_ [62] and D [63], which could significantly contribute to serum vitamin B_12_ and vitamin D levels.

Overall, we identified that plant-based, carnivore, and dairy and nondairy alternatives DPs were positively correlated with serum vitamin D levels and a reduced risk of low serum vitamin D. Serum vitamin D status could be affected not only by dietary patterns but also by exposure to sunlight or the use of sun protection [64]. Our previous study showed that among 1502 pregnant women in Taiwan, 69.6% women had sun exposure ≥10 min/d in the previous month, and 61.7% women had blood drawn in sunny months between June and November [65]. Additionally, dietary vitamin D intake had a greater impact on serum vitamin D levels in the women who lived in the northern part of Taiwan, whereas serum vitamin D levels were more greatly influenced by sunlight-related factors in those who lived in the southern or other parts of Taiwan [65]. These vitamin-D-associated DPs may reduce the risk of anemia in pregnant women, because these DPs were also negatively correlated with other anemia-related biochemical variables such as serum folate and vitamin B_12_. A possible mechanism for the effect of vitamin D on anemia was its modulation in iron metabolism via the down-regulation of hepcidin [66,67]. Higher serum vitamin D levels could be beneficial for better iron statuses through reducing hepcidin at the transcriptional level and suppressing the expression of proinflammatory cytokines involved in iron imbalance [67]. Active vitamin D could down-regulate the production of endogenous hormone hepcidin, thereby improving iron release, iron recycling, and iron absorption [67], and further maintain iron status during pregnancy. A recent cross-sectional study reported that serum hepcidin levels were negatively associated with the consumption frequency of plant-based foods such as legumes, breakfast cereals, light-colored vegetables, and gourds and root vegetables in Taiwanese pregnant women [25]. In the present study, we did not analyze serum hepcidin, and further studies are necessary to identify whether vitamin-D-rich DP is correlated with serum hepcidin levels.

### 4.3. Strengths and Limitations

To the best of our knowledge, the present study pioneered the PCA-mediated identification method for the association of DPs with serum levels of vitamin D and iron biomarkers in Taiwanese pregnant women. PCA is commonly used in pragmatic analyses performed using correlation matrices of intake units to identify common DPs [68]. We used data from the Pregnant NAHSIT 2017–2019 and included pregnant women from different areas of Taiwan (northern, central, southern, and eastern). In addition, sociodemographic data (education and income levels) were also collected to complement our findings.

The present study has some limitations. First, because of the unavailability of data regarding serum vitamin C, hepcidin, and parathyroid hormone levels which could affect iron status, we could not assess the association of DPs with these biomarkers. Second, the use of the FFQ and self-reported data for body weight and height might have introduced biases, such as errors in overestimation or underestimation. To overcome or minimize the biases of the FFQ, we additionally obtained 24 h dietary recall data and used food pictures and measurement cups or spoons during data collection [69]. Third, we did not consider certain pathological conditions of pregnant women, such as morning sickness during the first trimester of pregnancy. Fourth, the data regarding dietary supplements and seasonality were limited. Finally, because of the cross-sectional study design, we could not establish any causal relationship among DPs, serum vitamin D levels, and iron status. Nevertheless, a correlation relationship was identified between DPs and serum levels of anemia-related biomarkers. Future cohort studies and randomized control trials are needed to overcome the aforementioned limitations.

## 5. Conclusions

This study is a novel attempt to identify the associations among DPs, serum vitamin D levels, and iron status in pregnant women. Plant-based (DP-1), carnivore (DP-2), and dairy and nondairy alternatives DPs (DP-4) are positively correlated with serum vitamin D levels. The medium intake of a plant-based DP (DP-1) is associated with higher levels of serum folate and vitamin D. The medium and high consumption of carnivore DP (DP-2) is correlated with higher levels of serum vitamin B_12_ and vitamin D. The high intake of dairy and nondairy alternatives DP (DP-4) is associated with higher levels of serum folate and vitamin B_12_. However, we found no strong association between DPs and serum levels of Hb and iron status, except the negative correlation between the carnivore DP (DP-2) and serum iron levels. Thus, the medium intake of a vitamin D-rich diet such as a plant-based, carnivore, or dairy and nondairy alternatives DP is suggested to be beneficial for preventing anemia in pregnant women due to better statuses of serum folate, vitamin B_12_, and vitamin D.

## Figures and Tables

**Table 1:** Sociodemographic and anthropometric characteristics of the women across the tertiles of serum vitamin D (*n* = 1502) ^1^.

Variables	Total (*n*)	Tertiles of Serum Vitamin D ^2^			
		T1 (*n* = 505)	T2 (*n* = 486)	T3 (*n* = 511)	*p*-Value ^3^
**Sociodemographic Data**					
Age (years)	1502	32.0 ± 4.7 ^a^	32.7 ± 4.7 ^ab^	32.9 ± 4.9 ^b^	0.008
Region of residence	1499				0.000
Northern		231 (45.8)	153 (31.5)	117 (23.0)	
Central		130 (25.8)	124 (25.5)	117 (23.0)	
Southern		51 (10.1)	77 (15.8)	163 (32.0)	
Eastern and other		92 (18.3)	132 (27.2)	112 (22.0)	
Parity	1497				0.002
1		306 (60.7)	267 (55.2)	251 (49.3)	
2		164 (32.5)	170 (35.1)	193 (37.9)	
≥3		34 (6.8)	47 (9.7)	65 (12.8)	
Trimester	1502				0.000
First, weeks 0–12		172 (34.1)	125 (25.7)	78 (15.3)	
Second, weeks 13–26		164 (32.5)	159 (32.7)	162 (31.7)	
Third, weeks 27–40		169 (33.4)	202 (41.6)	271 (53.0)	
Education	1493				0.165
≤Junior high school		68 (13.5)	72 (15.0)	97 (19.1)	
College or university		355 (70.3)	330 (68.8)	340 (66.9)	
Graduate school		82 (16.2)	78 (16.2)	71 (14.0)	
Monthly income (NTD)	1474				0.117
<30,000		63 (12.6)	69 (14.8)	80 (15.8)	
30,000–59,999		209 (41.7)	187 (40.1)	238 (46.9)	
60,000–99,999		162 (32.3)	150 (32.2)	131 (25.8)	
≥100,000		67 (13.4)	60 (12.9)	58 (11.5)	
Sun exposure (min/d)	1498				0.676
<10		158 (31.3)	147 (30.4)	155 (30.5)	
10 to <20		155 (30.7)	150 (31.0)	144 (28.2)	
20 to <60		172 (34.0)	158 (32.6)	179 (35.2)	
≥60		20 (4.0)	29 (6.0)	31 (6.1)	
**Anthropometric Data**					
Pre-pregnant BMI (kg/m^2^)	1479	22.4 ± 3.9	22.9 ± 4.2	22.8 ± 4.0	0.082
<18.5		52 (10.5)	45 (9.4)	44 (8.7)	0.739
18.5 to <24.0		312 (62.9)	285 (59.6)	309 (61.2)	
24.0 to <27.0		71 (14.3)	76 (15.9)	84 (16.6)	
≥27		61 (12.3)	72 (15.1)	68 (13.5)	

^1^ Continuous data are presented as the mean ± standard deviation, whereas categorical data are presented as the number and percentage in the parentheses. Different superscript letters for continuous variables indicate significantly different (*p* ≤ 0.05) using Turkey’s post hoc test. ^2^ Tertiles of serum vitamin D levels: T1: 20 to >53 nmol/L, T2: 54 to >71 nmol/L, and T3: 72 to 154 nmol/L. ^3^ The *p*-value was determined using one-way analysis of variance test for continuous variables and chi-square test for categorical variables. BMI, body mass index.

**Table 2:** Biochemical characteristics of the women across the tertiles of serum vitamin D (*n* = 1502) ^1^.

Serum Variables	Tertiles of Serum Vitamin D ^2^			
	T1 (*n* = 505)	T2 (*n* = 486)	T3 (*n* = 511)	*p*-Value ^3^
Hemoglobin (mmol/L)	7.2 ± 1.1 ^a^	7.3 ± 1.2 ^ab^	7.4 ± 1.3 ^b^	0.038
<4.34	4 (0.8)	5 (1.0)	1 (0.2)	0.051
4.34–6.14	38 (7.5)	43 (8.9)	42 (8.2)	
6.15–6.76	80 (15.8)	50 (10.3)	59 (11.5)	
6.77–8.69	364 (72.1)	369 (75.9)	376 (73.6)	
>8.69	19 (3.8)	19 (3.9)	33 (6.5)	
Iron (µmol/L)	12.0 ± 6.8 ^a^	12.8 ± 6.6 ^a^	13.9 ± 7.8 ^b^	0.000
Ferritin (nmol/L)	0.06± 0.15 ^a^	0.05 ± 0.06 ^ab^	0.05 ± 0.05 ^b^	0.028
TIBC (µmol/L)	81.5 ± 19.9 ^a^	83.2 ± 17.2 ^ab^	85.6 ± 17.1 ^b^	0.001
Transferrin saturation (%)	16.2 ± 9.4	16.6 ± 10.1	16.7 ± 10.2	0.779
Folate (nmol/L)	25.5 ± 15.6 ^a^	29.2 ± 16.6 ^b^	32.3 ± 17.0 ^c^	0.000
<6.8	13 (2.6)	10 (2.0)	10 (2.0)	0.000
6.8–13.4	101 (20.0)	65 (13.4)	54 (10.6)	
13.5–45.3	342 (67.7)	346 (71.2)	349 (68.3)	
>45.3	49 (9.7)	65 (13.4)	98 (19.2)	
Vitamin B_12_ (pmol/L)	215.1 ± 154.9 ^a^	232.1 ± 108.9 ^ab^	249.0 ± 169.8 ^b^	0.001
Vitamin D (nmol/L)	42.4 ± 8.0 ^a^	62.3 ± 5.2 ^b^	89.1 ± 15.1 ^c^	0.000

^1^ Continuous data are presented as the mean ± standard deviation, whereas categorical data are presented as the number and percentage in the parentheses. Different superscript letters for continuous variables indicate significant difference (*p* ≤ 0.05) using Turkey’s post hoc test. ^2^ Tertiles of serum vitamin D levels: T1: 20 to >53 nmol/L, T2: 54 to >71 nmol/L, and T3: 72 to 154 nmol/L. ^3^ The *p*-value was determined using one-way analysis of variance test for continuous variables and chi-square test for categorical variables. TIBC, total iron-binding capacity.

**Table 3:** The association of plant-based dietary pattern with anemia-related biochemical variables in serum evaluated by the generalized linear regression analysis ^1^.

Variables	Model 1	Model 2	Model 3
	β (95% CI)	β (95% CI)	β (95% CI)
Hemoglobin (mmol/L)	−0.04 (−1.76, 1.50)	0.01 (−1.42, 1.85)	0.00 (−1.52, 1.13)
Iron (µmol/L)	−0.04 (−0.46, 0.08)	−0.03 (−0.45, 1.01)	−0.03 (−0.46, 0.09)
Ferritin (nmol/L)	−0.06 (−0.29, −0.01) *	−0.02 (−0.20, 0.07)	−0.02 (−0.19, 0.09)
TIBC (µmol/L)	0.09 (0.02, 0.10) ***	0.02 (−0.01, 0.03)	0.02 (−0.01, 0.03)
Transferrin saturation (%)	0.00 (−0.01, 0.01)	0.00 (−0.01, 0.01)	−0.00 (−0.01, 0.01)
Folate (nmol/L)	0.01 (−0.02, 0.03)	0.02 (−0.01, 0.03)	0.02 (−0.01, 0.03)
Vitamin B_12_ (pmol/L)	−0.04 (−0.36, 0.04)	−0.02 (−0.28, 0.11)	−0.02 (−0.27, 0.13)
Vitamin D (nmol/L)	0.08 (0.02, 0.08) **	0.06 (0.00, 0.06) *	0.04 (−0.00, 0.05) *

^1^ The values of β and data in the parentheses indicate regression coefficient and 95% confidence interval (95% CI), respectively, after covariate adjustment in different models: model 1, crude model; model 2, adjusted for age, region of residence, parity, and trimester; and model 3, adjusted for age, region of residence, parity, trimester, and daily dietary intake, such as energy (kcal), carbohydrate (% of energy), protein (g and % of energy), fat (g and % of energy), iron (mg), folate (µg), and vitamin D (µg). * *p* ≤ 0.05, ** *p* ≤ 0.01, and *** *p* ≤ 0.001. TIBC, total iron-binding capacity.

**Table 4:** The association of carnivore dietary pattern with anemia-related biochemical variables in serum evaluated by the generalized linear regression analysis ^1^.

Variables	Model 1	Model 2	Model 3
	β (95% CI)	β (95% CI)	β (95% CI)
Hemoglobin (mmol/L)	−0.03 (−1.82, 0.43)	−0.02 (−1.56, 0.74)	−0.02 (−1.60, 0.69)
Iron (µmol/L)	−0.08 (−0.49, −0.10) **	−0.07 (−0.47, −0.07) **	−0.08 (−0.50, −0.11) **
Ferritin (nmol/L)	−0.06 (−0.46, −0.04) *	−0.03 (−0.34, 0.06)	−0.03 (−0.32, 0.08)
TIBC (µmol/L)	0.08 (0.02, 0.10) **	0.02 (−0.01, 0.04)	0.02 (−0.01, 0.04)
Transferrin saturation (%)	0.01 (−0.02, 0.01)	−0.01 (−0.02, 0.01)	−0.02 (−0.02, 0.01)
Folate (nmol/L)	−0.02 (−0.05, 0.02)	−0.01 (−0.00, 0.00)	−0.00 (−0.03, 0.03)
Vitamin B_12_ (pmol/L)	0.00 (−0.29, 0.29)	0.02 (−0.18, 0.39)	0.01 (−0.21, 0.36)
Vitamin D (nmol/L)	0.08 (0.02, 0.10) **	0.06 (0.00, 0.08) *	0.04 (−0.00, 0.07) *

^1^ The values of β and data in the parentheses indicate regression coefficient and 95% confidence interval (95% CI), respectively, after covariate adjustment in different models: model 1, crude model; model 2, adjusted for age, region of residence, parity, and trimester; and model 3, adjusted for age, region of residence, parity, trimester, and daily dietary intake, such as energy (kcal), carbohydrate (% of energy), protein (g and % of energy), fat (g and % of energy), iron (mg), folate (µg), and vitamin D (µg). * *p* ≤ 0.05 and ** *p* ≤ 0.01. TIBC, total iron-binding capacity.

**Table 5:** The association of dairy and nondairy alternatives dietary pattern with anemia-related biochemical variables in serum evaluated by the generalized linear regression analysis ^1^.

Variables	Model 1	Model 2	Model 3
	β (95% CI)	β (95% CI)	β (95% CI)
Hemoglobin (mmol/L)	−0.03 (−1.75, 0.33)	−0.03 (−0.30, 0.06)	−0.03 (−1.70, 0.41)
Iron (µmol/L)	−0.04 (−0.29, 0.05)	−0.03 (−0.30, 0.06)	−0.04 (−0.32, 0.04)
Ferritin (nmol/L)	−0.04 (−0.41, 0.03)	−0.02 (−0.30, 0.12)	−0.04 (−0.31, 2.00)
TIBC (µmol/L)	0.08 (0.02, 0.10) **	0.02 (−0.01, 0.05)	0.02 (−0.01, 0.05)
Transferrin saturation (%)	0.01 (−0.02, 0.02)	0.01 (−0.02, 0.02)	0.01 (−0.02, 0.02)
Folate (nmol/L)	0.03 (−0.02, 0.05)	0.04 (−0.01, 0.06)	0.04 (−0.01, 0.06)
Vitamin B_12_ (pmol/L)	−0.01 (−0.38, 0.24)	0.01 (−0.25, 0.35)	0.01 (−0.27, 0.34)
Vitamin D (nmol/L)	0.05 (0.02, 0.09) *	0.04 (−0.00, 0.08) *	0.03 (−0.01, 0.07)

^1^ The values of β and data in the parentheses indicate regression coefficient and 95% confidence interval (95% CI), respectively, after covariate adjustment in different models: model 1, crude model; model 2, adjusted for age, region of residence, parity, and trimester; and model 3, adjusted for age, region of residence, parity, trimester, and daily dietary intake, such as energy (kcal), carbohydrate (% of energy), protein (g and % of energy), fat (g and % of energy), iron (mg), folate (µg), and vitamin D (µg). * *p* ≤ 0.05 and ** *p* ≤ 0.01. TIBC, total iron-binding capacity.

**Table 6:** Odds ratios (ORs) of low-anemia-related biochemical variables in serum across the tertiles of plant-based dietary pattern assessed by binomial logistic regression analysis ^1^.

Variables ^2^	Plant-Based Dietary Pattern ^3^					
	Model 1 OR (95% Confidence Interval)		Model 2 OR (95% Confidence Interval)		Model 3 OR (95% Confidence Interval)	
	T2	T3	T2	T3	T2	T3
Hemoglobin (mmol/L)	1.40 (0.44, 4.45)	1.00 (0.35, 2.87)	1.53 (0.47, 4.96)	0.94 (0.32, 2.77)	1.12 (0.32, 3.87)	0.65 (0.20, 2.04)
Iron (µmol/L)	0.98 (0.76, 1.25)	1.17 (0.91, 1.50)	0.98 (0.75, 1.27)	1.14 (0.88, 1.49)	0.96 (0.73, 1.25)	1.14 (0.87, 1.49)
Ferritin (nmol/L)	0.98 (0.76, 1.25)	0.73 (0.57, 0.94) *	0.92 (0.69, 1.22)	1.17 (0.87, 1.57)	0.89 (0.67, 1.20)	1.16 (0.86, 1.56)
TIBC (µmol/L)	1.00 (0.02, 1.28)	1.33 (0.04, 1.52)	1.07 (0.14, 1.48)	0.95 (0.12, 1.34)	1.06 (0.14, 1.48)	0.97 (0.12, 1.37)
Transferrin saturation (%)	1.06 (0.82, 1.36)	0.84 (0.65, 1.07)	1.04 (0.81, 1.34)	0.83 (0.65, 1.07)	1.03 (0.80, 1.33)	0.83 (0.64, 1.07)
Folate (nmol/L)	0.61 (0.44, 0.85) **	0.66 (0.48, 0.92) *	0.61 (0.42, 0.88) **	0.68 (0.47,0.98) *	0.60 (0.41, 0.87) **	0.69 (0.47, 1.00)
Vitamin B_12_ (pmol/L)	0.86 (0.64, 1.16)	1.01 (0.76, 1.35)	0.86 (0.64, 1.18)	0.95 (0.70, 1.29)	0.88 (0.67, 1.24)	1.04 (0.77, 1.42)
Vitamin D (nmol/L)	0.69 (0.52,0.92) *	0.75 (0.57, 0.99) *	0.68 (0.51, 0.91) *	0.81 (0.60, 1.08)	0.69 (0.52, 0.93) *	0.84 (0.62, 1.13)

^1^ Three different models were performed in binomial logistic regression analysis: model 1, crude model; model 2, adjusted for age, region of residence, parity, and trimester; and model 3, adjusted for age, region of residence, parity, trimester, and daily dietary intake, such as energy (kcal), carbohydrate (% of energy), protein (g and % of energy), fat (g and % of energy), iron (mg), folate (µg), and vitamin D (µg). ^2^ Variables were divided into two levels on the basis of cutoff values in serum: hemoglobin, 6.52 mmol/L (10.5 g/dL); iron, 10.7 µmol/L (60 µg/dL); ferritin, 0.034 nmol/L (15 ng/mL); TIBC, 42.96 µmol/L (240 µg/dL); transferrin saturation, 16%; folate, 13.6 nmol/L (6 ng/mL); vitamin B_12_, 149.8 pmol/L (203 pg/mL); and vitamin D, 75 nmol/L (30 ng/mL). ^3^ Dietary pattern scores were divided into tertiles: T1 (reference), 0.56–38.85; T2, >38.87–65.61; and T3 >65.85–436.82. * *p* ≤ 0.05 and ** *p* ≤ 0.01. TIBC, total iron-binding capacity.

**Table 7:** Odds ratios (ORs) of low-anemia-related biochemical variables in serum across the tertiles of carnivore dietary pattern assessed by binomial logistic regression analysis ^1^.

Variables ^2^	Carnivore Dietary Pattern ^3^					
	Model 1 OR (95% Confidence Interval)		Model 2 OR (95% Confidence Interval)		Model 3 OR (95% Confidence Interval)	
	T2	T3	T2	T3	T2	T3
Hemoglobin (mmol/L)	0.55 (0.18, 1.66)	1.00 (0.29, 3.48)	0.51 (0.17, 1.58)	1.03 (0.29, 3.68)	0.47 (0.14, 1.56)	0.93 (0.24, 3.54)
Iron (µmol/L)	1.36 (1.06,1.76) *	1.33 (1.03, 1.72) *	1.24 (0.95, 1.63)	1.33 (1.02, 1.74) *	1.30 (0.99, 1.72)	1.33 (1.02, 1.75) *
Ferritin (nmol/L)	1.29 (1.00, 1.66)	1.30 (1.00, 1.68)	1.24 (0.92, 1.66)	1.15 (0.86, 1.54)	1.24 (0.92, 1.67)	1.16 (0.86, 1.56)
TIBC (µmol/L)	0.83 (0.63, 1.08)	0.78 (0.59, 1.01)	0.92 (0.65, 1.28)	1.00 (0.14, 1.11)	1.00 (0.13, 1.27)	1.04 (0.14, 1.11)
Transferrin saturation (%)	0.86 (0.66, 1.10)	0.93 (0.72, 1.20)	0.84 (0.65, 1.08)	0.92 (0.71, 1.19)	0.70 (0.54, 0.91) **	0.93 (0.72, 1.21)
Folate (nmol/L)	1.18 (0.84, 1.68)	1.30 (0.92, 1.84)	1.15 (0.79, 1.69)	1.25 (0.85, 1.83)	1.15 (0.78, 1.70)	1.26 (0.86, 1.86)
Vitamin B_12_ (pmol/L)	0.85 (0.63, 1.14)	0.89 (0.67, 1.19)	0.56 (0.37, 0.84) **	0.54 (0.35, 0.82) **	0.68 (0.52, 0.90) **	0.25(0.17, 0.37) ***
Vitamin D (nmol/L)	0.69 (0.52, 0.92) *	0.55 (0.41, 0.74) ***	0.70 (0.52, 0.94) *	0.58 (0.43, 0.78) ***	0.70 (0.52, 0.95) **	0.59 (0.44, 0.80) **

^1^ Three different models were performed in binomial logistic regression analysis: model 1, crude model; model 2, adjusted for age, region of residence, parity, and trimester; and model 3, adjusted for age, region of residence, parity, trimester, and daily dietary intake, such as energy (kcal), carbohydrate (% of energy), protein (g and % of energy), fat (g and % of energy), iron (mg), folate (µg), and vitamin D (µg). ^2^ Variables were divided into two levels on the basis of cutoff values in serum: hemoglobin, 6.52 mmol/L (10.5 g/dL); iron, 10.7 µmol/L (60 µg/dL), ferritin, 0.034 nmol/L (15 ng/mL); TIBC, 42.96 µmol/L (240 µg/dL); transferrin saturation, 16%; folate, 13.6 nmol/L (6 ng/mL); vitamin B_12_, 149.8 pmol/L (203 pg/mL); and vitamin D, 75 nmol/L (30 ng/mL). ^3^ Dietary pattern scores were divided into tertiles: T1 (reference), 0.56–38.85; T2, >38.87–65.61; and T3 >65.85–436.82. * *p* ≤ 0.05, ** *p* ≤ 0.01, and *** *p* ≤ 0.001. TIBC, total iron-binding capacity.

**Table 8:** Odds ratios (ORs) of low-anemia-related biochemical variables in serum across the tertiles of dairy and nondairy alternatives dietary pattern assessed by binomial logistic regression analysis ^1^.

Variables ^2^	Dairy and Nondairy Alternatives Dietary Pattern ^3^					
	Model 1 OR (95% Confidence Interval)		Model 2 OR (95% Confidence Interval)		Model 3 OR (95% Confidence Interval)	
	T2	T3	T2	T3	T2	T3
Hemoglobin (mmol/L)	0.63 (0.24, 1.65)	0.69 (0.35, 1.67)	0.73 (0.27, 1.95)	1.12 (0.72, 1.83)	0.61 (0.21, 1.78)	0.98 (0.41, 1.89)
Iron (µmol/L)	0.83 (0.65, 1.07)	0.90 (0.70, 1.16)	0.81 (0.62, 1.06)	0.84 (0.64, 1.10)	0.83 (0.63, 1.08)	0.85 (0.65, 1.12)
Ferritin (nmol/L)	0.91 (0.71, 1.17)	1.11 (0.86, 1.42)	0.91 (0.71, 1.17)	1.11 (0.86, 1.42)	0.85 (0.64, 1.15)	0.87 (0.64, 1.17)
TIBC (µmol/L)	1.03 (0.14, 1.26)	0.71 (0.54, 0.93) *	1.10 (0.15, 1.30)	0.94 (0.12, 1.24)	1.00 (0.16, 1.31)	0.95 (0.12, 1.25)
Transferrin saturation (%)	1.04 (0.81, 1.33)	0.96 (0.75, 1.23)	1.02 (0.79, 1.31)	0.95 (0.74, 1.22)	1.02 (0.79, 1.31)	0.96 (0.74, 1.24)
Folate (nmol/L)	0.72 (0.52, 1.01)	0.75 (0.53, 1.04)	0.80 (0.55, 1.15)	0.67 (0.47, 0.97) *	0.80 (0.55, 1.16)	0.67 (0.46, 0.98) *
Vitamin B_12_ (pmol/L)	0.80 (0.60, 1.07)	0.73 (0.54, 0.97) *	0.79 (0.59, 1.07)	0.63 (0.46, 0.85) **	0.81 (0.60, 1.10)	0.66 (0.48, 0.90) *
Vitamin D (nmol/L)	0.79 (0.60, 1.05)	0.72 (0.54, 0.96) *	0.79 (0.59, 1.06)	0.72 (0.54, 0.97) *	0.80 (0.60, 1.08)	0.75 (0.55, 1.02)

^1^ Three different models were performed in binomial logistic regression analysis: model 1, crude model; model 2, adjusted for age, region of residence, parity, and trimester; and model 3, adjusted for age, region of residence, parity, trimester, and daily dietary intake, such as energy (kcal), carbohydrate (% of energy), protein (g and % of energy), fat (g and % of energy), iron (mg), folate (µg), and vitamin D (µg). ^2^ Variables were divided into two levels on the basis of cutoff values in serum: hemoglobin, 6.52 mmol/L (10.5 g/dL); iron, 10.7 µmol/L (60 µg/dL), ferritin, 0.034 nmol/L (15 ng/mL); TIBC, 42.96 µmol/L (240 µg/dL); transferrin saturation, 16%; folate, 13.6 nmol/L (6 ng/mL); vitamin B_12_, 149.8 pmol/L (203 pg/mL); and vitamin D, 75 nmol/L (30 ng/mL). ^3^ Dietary pattern scores were divided into tertiles: T1 (reference), 0.56–38.85; T2, >38.87–65.61; and T3 >65.85–436.82. * *p* ≤ 0.05 and ** *p* ≤ 0.01. TIBC, total iron-binding capacity.

## Data Availability

Data supporting the study findings are available from the database of Nationwide Nutrition and Health Survey in Pregnant Women in Taiwan. The data should be used for research purposes only. The study data are not publicly available.

## Supplementary Materials

The following supporting information can be downloaded at: https://www.mdpi.com/article/10.3390/nu15081805/s1. Table S1: Food groups and subgroups for dietary assessment; Table S2: Daily dietary intake of women across the tertiles of serum vitamin D levels (*n* = 1502); Table S3: The association of processed food dietary pattern with anemia-related biochemical variables in serum evaluated by the generalized linear regression analysis; Table S4: Odds ratios (ORs) of low-anemia-related biochemical variables in serum across the tertiles of processed food dietary pattern assessed by binomial logistic regression analysis.

## References

[1] Shah T., Khaskheli M.S., Ansari S., Lakhan H., Shaikh F., Zardari A.A., Warsi J., Rind N.A., Rind K.H., Shar A.H. (2022). Gestational anemia and its effects on neonatal outcome, in the population of Hyderabad, Sindh, Pakistan. Saudi J. Biol. Sci..

[2] Imai K. (2020). Parity-based assessment of anemia and iron deficiency in pregnant women. J. Obstet. Gynecol..

[3] World Health Organization (2011). Haemoglobin Concentrations for the Diagnosis of Anaemia and Assessment of Severity.

[4] Centers for Disease Control (1989). CDC criteria for anemia in children and childbearing-aged women. MMWR Morb. Mortal. Wkly. Rep..

[5] Anlaakuu P., Anto F. (2017). Anaemia in pregnancy and associated factors: A cross sectional study of antenatal attendants at the Sunyani Municipal Hospital, Ghana. BMC Res. Notes.

[6] Wedderburn C.J., Ringshaw J.E., Donald K.A., Joshi S.H., Subramoney S., Fouche J.-P., Stadler J.A.M., Barnett W., Rehman A.M., Hoffman N. (2022). Association of maternal and child anemia with brain structure in early life in South Africa. JAMA Netw. Open.

[7] Uta M., Neamtu R., Bernad E., Mocanu A.G., Gluhovschi A., Popescu A., Dahma G., Dumitru C., Stelea L., Citu C. (2022). The influence of nutritional supplementation for iron deficiency anemia on pregnancies associated with SARS-CoV-2 infection. Nutrients.

[8] Elema T.B., Yimam K.B., Waka F.C., Olana B.N. (2018). Folate and vitamin B-12 status of anemic pregnant women and association to hemoglobin during antenatal care, 17–37 weeks in Ambo Hospital, Oromia, Ethiopia, a multi regression analysis of socio-economic and serum folate and vitamin B-12. J. Nutr. Hum. Health.

[9] Behere R.V., Deshmukh A.S., Otiv S., Gupte M.D., Yajnik C.S. (2021). Maternal vitamin B12 status during pregnancy and its association with outcomes of pregnancy and health of the offspring: A systematic review and implications for policy in India. Front. Endocrinol..

[10] Finkelstein J.L., Fothergill A., Krisher J.T., Thomas T., Kurpad A.V., Dwarkanath P. (2021). Maternal vitamin B12 deficiency and perinatal outcomes in Southern India. PLoS ONE.

[11] Greenberg J.A., Bell S.J., Guan Y., Yu Y.H. (2011). Folic acid supplementation and pregnancy: More than just neural tube defect prevention. Rev. Obstet. Gynecol..

[12] Warner M.J., Kamran M.T. (2017). Iron Deficiency Anemia.

[13] Diana A., Purnamasari D.M., Rahmannia S., Luftimas D.E., Haszard J.J., Gibson R.S., Houghton L.A. (2019). Multimicronutrient biomarkers are related to anemia during infancy in Indonesia: A repeated cross-sectional study. Curr. Dev. Nutr..

[14] Daru J., Colman K., Stanworth S.J., De La Salle B., Wood E.M., Pasricha S.R. (2017). Serum ferritin as an indicator of iron status: What do we need to know?. Am. J. Clin. Nutr..

[15] Crispin P., Stephens B., McArthur E., Sethna F. (2019). First trimester ferritin screening for pre-delivery anaemia as a patient blood management strategy. Transfus. Apher. Sci..

[16] Rahman S.M., Siraj M.S., Islam M.R., Rahman A., Ekström E.C. (2021). Association between maternal plasma ferritin level and infants’ size at birth: A prospective cohort study in rural Bangladesh. Glob. Health Action.

[17] Abu-Ouf N.M., Jan M.M. (2015). The impact of maternal iron deficiency and iron deficiency anemia on child’s health. Saudi Med. J..

[18] Li N., Zhao G., Wu W., Zhang M., Liu W., Chen Q., Wang X. (2020). The efficacy and safety of vitamin C for iron supplementation in adult patients with iron deficiency anemia: A randomized clinical trial. JAMA Netw. Open.

[19] Heffernan A., Evans C., Holmes M., Moore J.B. (2017). The regulation of dietary iron bioavailability by vitamin C: A systematic review and meta-analysis. Proc. Nutr. Soc..

[20] Mogire R.M., Muriuki J.M., Morovat A., Mentzer A.J., Webb E.L., Kimita W., Ndungu F.M., Macharia A.W., Cutland C.L., Sirima S.B. (2022). Vitamin D deficiency and its association with iron deficiency in African children. Nutrients.

[21] Smith E.M., Tangpricha V. (2015). Vitamin D and anemia: Insights into an emerging association. Curr. Opin. Endocrinol. Diabetes Obes..

[22] Arabi S.M., Ranjbar G., Bahrami L.S., Vafa M., Norouzy A. (2020). The effect of vitamin D supplementation on hemoglobin concentration: A systematic review and meta-analysis. Nutr. J..

[23] Qiu F., Li R., Gu S., Zhao Y., Yang L. (2022). The effect of iron dextran on vitamin D_3_ metabolism in SD rats. Nutr. Metab..

[24] Si S., Peng Z., Cheng H., Zhuang Y., Chi P., Alifu X., Zhou H., Mo M., Yu Y. (2022). Association of vitamin D in different trimester with hemoglobin during pregnancy. Nutrients.

[25] Mayasari N.R., Bai C.H., Hu T.Y., Chao J.C., Chen Y.C., Huang Y.L., Wang F.F., Tinkov A.A., Skalny A.V., Chang J.S. (2021). Associations of food and nutrient intake with serum hepcidin and the risk of gestational iron-deficiency anemia among pregnant women: A population-based study. Nutrients.

[26] Michalski E.S., Nguyen P.H., Gonzalez-Casanova I., Nguyen S.V., Martorell R., Tangpricha V., Ramakrishnan U. (2017). Serum 25-hydroxyvitamin D but not dietary vitamin D intake is associated with hemoglobin in women of reproductive age in rural Northern Vietnam. J. Clin. Transl. Endocrinol..

[27] Wong R.S., Tung K.T.S., Chan Y.W.K., Chan B.N.K., Leung W.C., Yam J.C., Ip P. (2022). Adequate dietary intake and vitamin D supplementation: A study of their relative importance in determining serum vitamin D and ferritin concentrations during pregnancy. Nutrients.

[28] Zhang F., Tapera T.M., Gou J. (2018). Application of a new dietary pattern analysis method in nutritional epidemiology. BMC Med. Res. Methodol..

[29] Zang J., Luo B., Chang S., Jin S., Shan C., Ma L., Zhu Z., Guo C., Zou S., Jia X. (2019). Validity and reliability of a food frequency questionnaire for assessing dietary intake among Shanghai residents. Nutr. J..

[30] Schwedhelm C., Iqbal K., Knüppel S., Schwingshackl L., Boeing H. (2018). Contribution to the understanding of how principal component analysis–derived dietary patterns emerge from habitual data on food consumption. Am. J. Clin. Nutr..

[31] Jolliffe I.T., Cadima J. (2016). Principal component analysis: A review and recent developments. Philos. Trans. A Math. Phys. Eng. Sci..

[32] Pfeiffer C.M., Looker A.C. (2017). Laboratory methodologies for indicators of iron status: Strengths, limitations, and analytical challenges. Am. J. Clin. Nutr..

[33] Yamanishi H., Iyama S., Yamaguchi Y., Kanakura Y., Iwatani Y. (2003). Total iron-binding capacity calculated from serum transferrin concentration or serum iron concentration and unsaturated iron-binding capacity. Clin. Chem..

[34] Shane B. (2011). Folate status assessment history: Implications for measurement of biomarkers in NHANES. Am. J. Clin. Nutr..

[35] Karmi O., Zayed A., Baraghethi S., Qadi M., Ghanem R. (2011). Measurement of vitamin B12 concentration: A review on available methods. IIOAB J..

[36] Abdel-Wareth L., Haq A., Turner A., Khan S., Salem A., Mustafa F., Hussein N., Pallinalakam F., Grundy L., Patras G. (2013). Total vitamin D assay comparison of the Roche Diagnostics “Vitamin D total” electrochemiluminescence protein binding assay with the Chromsystems HPLC method in a population with both D2 and D3 forms of vitamin D. Nutrients.

[37] Health Promotion Administration, Ministry of Health and Welfare (2018). Taiwan’s Obesity Prevention and Management Strategy.

[38] Bellanger R.A. (2010). Iron deficiency anemia in women. US Pharm..

[39] Sukla S.K., Mohanty P.K., Patel S., Das K., Hiregoudar M., Soren U.K., Meher S. (2021). Iron profile of pregnant sickle cell anemia patients in Odisha, India. Hematol. Transfus. Cell Ther..

[40] Word Health Organization (2020). WHO Guideline on Use of Ferritin Concentrations to Assess Iron Status in Individuals and Populations.

[41] World Health Organization (2001). Archived: Iron Deficiency Anemia: Assessment, Prevention and Control.

[42] World Health Organization (2015). Serum and Red Blood Cell Folate Concentrations for Assessing Folate Status in Populations. Vitamin and Mineral Nutrition Information System.

[43] Holick M.F., Binkley N.C., Bischoff-Ferrari H.A., Gordon C.M., Hanley D.A., Heaney R.P., Murad M.H., Weaver C.M., Endocrine Society (2011). Evaluation, treatment, and prevention of vitamin D deficiency: An Endocrine Society clinical practice guideline. J. Clin. Endocrinol. Metab..

[44] Kurniawan A., Hsu C.-Y., Rau H., Lin L.-Y., Chao J. (2019). Dietary patterns in relation to testosterone levels and severity of impaired kidney function among middle-aged and elderly men in Taiwan: A cross-sectional study. Nutr. J..

[45] Szumilas M. (2010). Explaining odds ratios. J. Can. Acad. Child Adolesc. Psychiatry.

[46] Judistiani R.T.D., Gumilang L., Nirmala S.A., Irianti S., Wirhana D., Permana I., Sofjan L., Duhita H., Tambunan L.A., Gurnadi J.I. (2018). Association of colecalciferol, ferritin, and anemia among pregnant women: Result from cohort study on vitamin D status and its impact during pregnancy and childhood in Indonesia. Anemia.

[47] Perzia B.M., Ying G.-S., Dunaief J.L., Dunaief D.M. (2022). Reduction in ferritin concentrations among patients consuming a dark-green leafy vegetable-rich, low inflammatory foods everyday (LIFE) diet. Curr. Dev. Nutr..

[48] Ma Q., Kim E.-Y., Lindsay E., Han O. (2011). Bioactive dietary polyphenols inhibit heme iron absorption in a dose-dependent manner in human intestinal Caco-2 cells. J. Food Sci..

[49] Piskin E., Cianciosi D., Gulec S., Tomas M., Capanoglu E. (2022). Iron absorption: Factors, limitations, and improvement methods. ACS Omega.

[50] Koebnick C., Heins U.A., Hoffmann I., Dagnelie P.C., Leitzmann C. (2001). Folate status during pregnancy in women is improved by long-term high vegetable intake compared with the average western diet. J. Nutr..

[51] Specker B.L., Tsang R.C., Ho M., Miller D. (1987). Effect of vegetarian diet on serum 1,25-dihydroxyvitamin D concentrations during lactation. Obstet. Gynecol..

[52] Bhatnagar R.S., Padilla-Zakour O.I. (2021). Plant-based dietary practices and socioeconomic factors that influence anemia in India. Nutrients.

[53] Pawlak R., Berger J., Hines I. (2018). Iron status of vegetarian adults: A review of literature. Am. J. Lifestyle Med..

[54] Jackson J., Williams R., McEvoy M., MacDonald-Wicks L., Patterson A. (2016). Is higher consumption of animal flesh foods associated with better iron status among adults in developed countries? A systematic review. Nutrients.

[55] Tuntipopipat S., Zeder C., Siriprapa P., Charoenkiatkul S. (2009). Inhibitory effects of spices and herbs on iron availability. Int. J. Food Sci. Nutr..

[56] Broderstad A.R., Melhus M., Brustad M., Lund E. (2011). Iron stores in relation to dietary patterns in a multiethnic population: The SAMINOR study. Public Health Nutr..

[57] Denissen K.F.M., Heil S.G., Eussen S.J.P.M., Heeskens J.P.J., Thijs C., Mommers M., Smits L.J.M., van Dongen M.C.J.M., Dagnelie P.C. (2019). Intakes of vitamin B-12 from dairy food, meat, and fish and shellfish are independently and positively associated with vitamin B-12 biomarker status in pregnant Dutch women. J. Nutr..

[58] McGowan C.A., Byrne J., Walsh J., McAuliffe F.M. (2011). Insufficient vitamin D intakes among pregnant women. Eur. J. Clin. Nutr..

[59] Gille D., Schmid A. (2015). Vitamin B12 in meat and dairy products. Nutr. Rev..

[60] Paula W.O., Gonçalves V.S.S., Patriota E.S.O., Franceschini S.C.C., Pizato N. (2023). Impact of ultra-processed food consumption on quality of diet among Brazilian pregnant women assisted in primary health care. Int. J. Environ. Res. Public Health.

[61] Cifelli C.J., Agarwal S., Fulgoni V.L. (2022). Association between intake of total dairy and individual dairy foods and markers of folate, vitamin B_6_ and vitamin B_12_ status in the U.S. Population. Nutrients.

[62] Matte J.J., Britten M., Girard C.L. (2014). The importance of milk as a source of vitamin B_12_ for human nutrition. Anim. Front..

[63] Polzonetti V., Pucciarelli S., Vincenzetti S., Polidori P. (2020). Dietary intake of vitamin D from dairy products reduces the risk of osteoporosis. Nutrients.

[64] Dasgupta A., Saikia U., Sarma D. (2012). Status of 25(OH)D levels in pregnancy: A study from the North Eastern part of India. Indian J. Endocrinol. Metab..

[65] Huang Y.-L., Pham T.T.M., Chen Y.-C., Chang J.-S., Chao J.C.-J., Bai C.-H. (2023). Effects of climate, sun exposure, and dietary intake on vitamin D concentrations in pregnant women: A population-based study. Nutrients.

[66] Pagani A., Nai A., Silvestri L., Camaschella C. (2019). Hepcidin and anemia: A tight relationship. Front. Physiol..

[67] Moran-Lev H., Weisman Y., Cohen S., Deutsch V., Cipok M., Bondar E., Lubetzky R., Mandel D. (2018). The interrelationship between hepcidin, vitamin D, and anemia in children with acute infectious disease. Pediatr. Res..

[68] Thorpe M.G., Milte C.M., Crawford D., McNaughton S.A. (2016). A comparison of the dietary patterns derived by principal component analysis and cluster analysis in older Australians. Int. J. Behav. Nutr. Phys. Act..

[69] Lyu L.C., Lin C.F., Chang F.H., Chen H.F., Lo C.C., Ho H.F. (2007). Meal distribution, relative validity and reproducibility of a meal-based food frequency questionnaire in Taiwan. Asia Pac. J. Clin. Nutr..

