# Supplementary material for: Associations of Dietary Patterns and Vitamin D Levels with Iron Status in Pregnant Women: A Cross-Sectional Study in Taiwan
Source: Nutrients. 2023 Apr 7;15(8):1805. doi: 10.3390/nu15081805 (PMC10143533; doi:10.3390/nu15081805)
Supplement: Supplementary file 1 [file nutrients-15-01805-s001.zip › nutrients-2265495-supplementary.pdf]

Supplementary Tables

Table S1. Food groups and subgroups for dietary assessment.

Food Groups	Subgroups
FFQ-G1	Dry rice and porridge
FFQ-G2	Noodles and related products
FFQ-G3	Breakfast cereals and bread and related products
FFQ-G4	Cake, pastry, and dumplings
FFQ-G5	Salty buns and sweet buns
FFQ-G6	Glutinous rice desserts and rhizome starch
FFQ-G7	Carrots, roots, and tubers
FFQ-G8	Eggs
FFQ-G9	Milk and milk products
FFQ-G10	Nondairy products, such as soy and rice milk
FFQ-G11	Light-colored vegetables
FFQ-G12	Dark-colored vegetables
FFQ-G13	Bamboo shoots and melons
FFQ-G14	Mushrooms and related products
FFQ-G15	Legumes and various beans
FFQ-G16	Marine plants and kelp
FFQ-G17	Pickled vegetables
FFQ-G18	Herbs and spices
FFQ-G19	General soy products and gluten pasta
FFQ-G20	Nuts and nut products
FFQ-G21	Fresh fruits
FFQ-G22	100% Pure juice and commercially available vegetable juice
FFQ-G23	Canned fruits, dehydrated fruits, pickled fruits, and jam
FFQ-G24	Aquatic fish, shell, shellfish, and seafood
FFQ-G25	Deep water fish
FFQ-G26	Seafood products
FFQ-G27	Poultry meat
FFQ-G28	Livestock lean meat
FFQ-G29	Livestock semifat meat
FFQ-G30	Animal organ meat and blood
FFQ-G31	Processed meat and meat pulp
FFQ-G32	Salt

FFQ, food frequency questionnaire.

Table S2. Daily dietary intake of women across the tertiles of serum vitamin D levels (*n* = 1502)[1].

Dietary Intake	Tertiles of Serum Vitamin D[2]			
	**T1 (*n* = 505)**	**T2 (*n* = 486)**	**T3 (*n* = 511)**	***p*-Value[3]**
Daily dietary nutrient intake (*n* = 1501)				
Energy (kcal)	1845.8 ± 654.6[ab]	1799.0 ± 562.1[a]	1898.4 ± 546.4[b]	0.029
Carbohydrate (g)	233.8 ± 88.1[a]	224.0 ± 81.2[a]	227.9 ± 76.8[a]	0.165
Carbohydrate (% of energy)	51.1 ± 9.7[a]	50.0 ± 9.8[a]	48.1 ± 9.8[b]	0.000
Protein (g)	68.5 ± 28.3[a]	67.2 ± 25.8[a]	74.0 ± 25.2[b]	0.000
Protein (% of energy)	14.5 ± 3.7[a]	15.0 ± 3.8[b]	14.8 ± 3.6[a]	0.002
Fat (g)	72.3 ± 34.4[a]	71.8 ± 30.2[a]	78.8 ± 32.4[b]	0.001
Fat (% of energy)	34.4 ± 9.0[a]	34.9 ± 9.0[a]	37.0 ± 8.9[b]	0.000
Iron (mg)	10.3 ± 6.6[a]	10.1 ± 5.2[a]	11.4 ± 8.7[b]	0.010
Folate (µg)	199.3 ± 92.4[a]	208.9 ± 112.8[ab]	216.0 ± 92.6[b]	0.030
Vitamin B_{12} (µg)	5.0 ± 9.5[a]	5.2 ± 7.7[a]	5.9 ± 8.3[a]	0.235
Vitamin D (µg)	7.7 ± 13.6[a]	8.5 ± 13.7[ab]	9.8 ± 14.5[b]	0.049
Supplement intake (frequency/month)				
Milk powder, 265 (17.6%)	-	-	-	-
Multivitamin/multimineral, 926 (61.7%)	2.5 ± 0.5[a]	2.7 ± 0.4[b]	2.8 ± 0.4[c]	0.000
Iron, 168 (11.2%)	-	-	-	-
Folate, 683 (45.5%)	2.6 ± 0.5[a]	2.6 ± 0.5[a]	2.7 ± 0.4[b]	0.007
Vitamin B complex, 270 (18.0%)	-	-	-	-
Vitamin D, 167 (11.1%)	-	-	-	-
Calcium, 660 (43.9%)	2.4 ± 0.5[a]	2.5 ± 0.5[b]	2.6 ± 0.5[b]	0.000

[1] Continuous data are presented as the mean ± standard deviation, whereas categorical data are presented as the number and percentage in the parentheses. Different superscript letters for continuous variables indicate significantly different ($p \leq 0.05$) using Turkey's post hoc test. The dash line indicates no data provided because less proportion (<20%) of the women took this supplement.

[2] Tertiles of serum vitamin D levels: T1: 20 to >53 nmol/L, T2: 54 to >71 nmol/L, and T3: 72 to 154 nmol/L.

[3] The *p*-value was determined using one-way analysis of variance test for continuous variables and chi-square test for categorical variables.

Table S3. The association of processed food dietary pattern with anemia related biochemical variables in serum evaluated by the generalized linear regression analysis[1].

Variables	Model 1	Model 2	Model 3
	β (95% CI)	β (95% CI)	β (95% CI)
Hemoglobin (mmol/L)	−0.00 (−0.43, 0.40)	−0.00 (−0.45, 0.40)	0.00 (−0.40, 0.43)
Iron (μmol/L)	0.03 (−0.03, 0.11)	0.02 (−0.04, 0.10)	0.03 (−0.03, 0.11)
Ferritin (nmol/L)	−0.01 (−0.56, 0.54)	−0.00 (−0.56, 0.50)	0.00 (−0.46, 0.62)
TIBC (μmol/L)	−0.03 (−0.14, 0.07)	−0.02 (−0.11, 0.05)	−0.03 (−0.13, 0.02)
Transferrin saturation (%)	−0.04 (−0.10, 0.01)	−0.04 (−0.10, 0.01)	−0.05 (−0.11, 0.00)
Folate (nmol/L)	0.03 (−0.04, 0.14)	0.02 (−0.05, 0.11)	0.02 (−0.05, 0.12)
Vitamin B_{12} (pmol/L)	−0.04 (−1.44, 0.09)*	−0.05 (−1.48, 0.02)*	−0.04 (−1.34, 0.18)
Vitamin D (nmol/L)	0.03 (−0.04, 0.20)	0.02 (−0.07, 0.16)	0.02 (−0.06, 0.18)

[1]The values of β and data in the parentheses indicate regression coefficient and 95% confidence interval (95% CI), respectively, after covariate adjustment in different models: model 1, crude model; model 2, adjusted for age, region of residence, parity, and trimester; and model 3, adjusted for age, region of residence, parity, trimester, and daily dietary intake, such as energy (kcal), carbohydrate (% of energy), protein (g and % of energy), fat (g and % of energy), iron (mg), folate (μg), and vitamin D (μg). *$p \leq 0.05$.
TIBC, total iron-binding capacity.

Table S4. Odds ratios (ORs) of low anemia related biochemical variables in serum across the tertiles of processed food dietary pattern assessed by binomial logistic regression analysis[1].

Variables[2]	Processed Food Dietary Pattern[3]					
	Model 1 OR (95% Confidence Interval)		Model 2 OR (95% Confidence Interval)		Model 3 OR (95% Confidence Interval)	
	T2	T3	T2	T3	T2	T3
Hemoglobin (mmol/L)	0.43 (0.11, 1.66)	0.33 (0.09, 1.23)	0.40 (0.15, 2.58)	0.94 (0.32, 2.77)	0.32 (0.07, 1.43)	0.69 (0.41, 1.17)
Iron (μmol/L)	1.04 (0.81, 1.33)	1.02 (0.79, 1.31)	1.00 (0.75, 1.29)	1.03 (0.79, 1.34)	1.05 (0.81, 1.37)	1.02 (0.78, 1.34)
Ferritin (nmol/L)	0.95 (0.74, 1.22)	0.98 (0.77, 1.26)	0.89 (0.67, 1.19)	0.88 (0.66, 1.18)	0.87 (0.65, 1.16)	0.82 (0.61, 1.11)
TIBC (μmol/L)	1.00 (0.14, 1.12)	0.90 (0.13, 1.11)	1.01 (0.15, 1.13)	1.00 (0.13, 1.13)	1.12 (0.16, 1.15)	1.02 (0.14, 1.12)
Transferrin saturation (%)	0.96 (0.75, 1.23)	1.07 (0.84, 1.38)	0.95 (0.74, 1.22)	1.07 (0.84, 1.38)	0.94 (0.73, 1.21)	1.09 (0.84, 1.41)
Folate (nmol/L)	0.73 (0.52, 1.01)	0.76 (0.54, 1.05)	0.78 (0.54, 1.13)	0.93 (0.64, 1.35)	0.76 (0.52, 1.11)	0.89 (0.61, 1.31)
Vitamin B_{12} (pmol/L)	0.89 (0.66, 1.20)	1.15 (0.86, 1.53)	0.91 (0.67, 1.24)	1.23 (0.91, 1.66)	0.89 (0.65, 1.22)	1.21 (0.89, 1.65)
Vitamin D (nmol/L)	0.71 (0.53, 0.95)*	0.97 (0.73, 1.28)	0.68 (0.51, 0.92)*	1.02 (0.76, 1.36)	0.75 (0.55, 1.02)	1.01 (0.75, 1.36)

[1] Three different models were performed in binomial logistic regression analysis: model 1, crude model; model 2, adjusted for age, region of residence, parity, and trimester; and model 3, adjusted for age, region of residence, parity, trimester, and daily dietary intake, such as energy (kcal), carbohydrate (% of energy), protein (g and % of energy), fat (g and % of energy), iron (mg), folate (μg), and vitamin D (μg).

[2] Variables were divided into two levels on the basis of cutoff values in serum: hemoglobin, 6.52 mmol/L (10.5 g/dL); iron, 10.7 μmol/L (60 μg/dL), ferritin, 0.034 nmol/L (15 ng/mL); TIBC, 42.96 μmol/L (240 μg/dL); transferrin saturation, 16%; folate, 13.6 nmol/L (6 ng/mL); vitamin B_{12}, 149.8 pmol/L (203 pg/mL); and vitamin D, 75 nmol/L (30 ng/mL).

[3] Dietary pattern scores were divided into tertiles: T1 (reference), 0.56-38.85; T2, >38.87-65.61; and T3 >65.85-436.82.

*$p \leq 0.05$.

TIBC, total iron-binding capacity.
